# Glucocorticoid evoked upregulation of RCAN1-1 in human leukemic CEM cells susceptible to apoptosis

**DOI:** 10.1186/1750-2187-4-6

**Published:** 2009-09-02

**Authors:** Yasuko Hirakawa, Laura J Nary, Rheem D Medh

**Affiliations:** 1 Department of Biology, California State University Northridge, Northridge, CA 91330-8303, USA

## Abstract

**Background:**

Glucocorticoid hormones (GCs) induce apoptosis of leukemic T-cells by transcriptional regulation via the GC receptor, GR. In the human leukemic CEM cell culture model, *RCAN1 *has been identified as one of the genes that is specifically upregulated only in the GC-sensitive CEM C7-14 cells, but not in the GC-resistant CEM-C1-15 sister cells in correlation with GC-evoked apoptosis. *RCAN1 *gene encodes two major protein isoforms of the regulator of calcineurin (RCAN1), RCAN1-1 and RCAN1-4 via alternative splicing of exons 1 and 4 respectively, to exons 5-7. Studies reported here evaluated the differential regulation and function of the two transcripts and protein products of RCAN1 by the synthetic GC dexamethasone (Dex), and by modulators of calcium signaling.

**Results:**

Dex selectively upregulates transcript specific for *RCAN 1-1 *in glucocorticoid (GC)-susceptible human leukemic CEM-C7-14 cells but not in GC-refractory CEM-C1-15 sister cells. Expression of the second major transcript, *RCAN1-4*, is upregulated by [Ca^2+^]_*i *_inducers, thapsigargin and A23187, but not by Dex, suggesting a mutually exclusive regulatory pathway for both *RCAN1 *transcripts. GC-mediated upregulation of *RCAN1-1 *transcript and RCAN1-1 protein was kinase dependent, and was blocked by staurosporine and the p38 MAP kinase inhibitor SB 202190. RCAN1-1 coimmunoprecipitates with calcineurin PP3C and Dex-mediated RCAN1-1 upregulation correlated with reduction in calcineurin PP3C activity.

**Conclusion:**

Data presented here suggest that GCs specifically upregulate *RCAN1-1 *transcript and protein while inducers of [Ca^2+^]_*i *_selectively upregulate *RCAN1-4*. GC-mediated increase in RCAN1-1 abundance and binding possibly inhibits calcineurin activity and modulates apoptosis in CEM-C7-14 cells.

## Background

Glucocorticoids (GCs) are effective antileukemic agents because of their ability to induce growth arrest and evoke apoptosis of normal thymocytes, immature peripheral T cells and many leukemic cells [1,2]. GCs activate the GC receptor (GR), a transcription factor that regulates expression of genes involved in modulating GC-induced actions such as immunosuppression, anti-inflammation and apoptosis [3]. Several laboratories have analyzed changes in gene expression profiles induced by GCs in an effort to identify candidate genes modulating GC-evoked apoptosis of leukemic lymphoid cells. Microarray analysis of a pair of GC-sensitive and -resistant human leukemic T-cell sister clones, CEM-C7-14 and CEM-C1-15, respectively, has shown that *RCAN1 *(Regulator of Calcineurin 1) gene, (also called *Adapt78 *or *DSCR1*) [4,5], whose protein product is RCAN1 (also called Calcipressin1), is one of the genes selectively upregulated by GCs only in CEM-C7-14 cells [6,7]. RCAN1 has been shown to bind to and modulate the activity of the catalytic subunit of the calcium-dependent phosphatase, calcineurin (PP3C) [4,8]. Calcineurin plays an important role in regulation of T-cell activation and apoptosis [9,10].

The *RCAN1 *locus is at chromosome 21q22.12 in humans, close to the Down Syndrome Critical region, and has been implicated in the pathophysiology of Down Syndrome and Alzheimer's disease [11,13], has been shown to regulate vascular function [14] and has been proposed to play an important role in the brain [15]. Alternative splicing and/or promoter usage results in multiple isoforms from the seven exons that make up the *RCAN1 *gene. The two major isoforms identified in most tissues are designated isoform 1 (exons 1+ 5-7; RCAN1-1) and isoform 4 (exons 4 + 5-7; RCAN1-4) [16]. These isoforms may be differentially upregulated in response to various stress signals, including calcium [17,18]. Calcineurin-dependent upregulation of *RCAN1-4 *(isoform 4) has been reported to occur through an alternative promoter in intron 3, which includes a cluster of 15 NFAT binding sites [19]. *RCAN1-1 *expression is upregulated in response to oxidant- and Ca^2+^-induced stress [20,21], and various reports suggest either a cytoprotective [20] or apoptotic [22,23] function for it. A recent knock out mouse model suggests that RCAN1 functions as a facilitator of calcineurin activity *in vivo *[24].

Phosphorylation of these isoforms at conserved serine residues has been shown to regulate their activity [25,27]. Unphosphorylated RCAN1 has been reported to bind to and inhibit calcineurin activity, serving as a feedback inhibitor of calcium signaling [12], while phosphorylation of RCAN1 has been shown to render it incapable of binding to calcineurin, thereby increasing calcineurin activity [27,28]. Indeed, phosphorylation of one or both serine residues in a 13-amino acid synthetic peptide attenuated *in vitro *inhibition of calcineurin activity [17]. Paradoxically, exogenous or over expressed RCAN1-1 phosphorylation has been shown to enhance calcineurin binding and inhibition [26]. Thus RCAN1 has been reported to both positively and negatively regulate calcineurin activity in various models. RCAN1 phosphorylation has also been shown to block its degradation and increase soluble and insoluble levels of RCAN1 in neuronal cells [29]

In studies presented here, we show that transcript and protein levels of isoform 1 of *RCAN1 *are selectively upregulated by GCs, and that GCs promote accumulation of an apparently unphosphorylated form of RCAN1-1, which is capable of binding calcineurin. Since GC mediated changes in RCAN1 expression and PP3C activity are restricted to the apoptosis-susceptible CEM subclone CEM-C7-14, and do not occur in the GC-resistant subclone CEM-C1-15, we deduce that RCAN1-1 may modulate GC-dependent calcineurin activity and GC- evoked apoptosis of leukemic lymphocytes.

## Results

Microarray analysis and Northern hybridization studies have previously suggested GC-dependent upregulation of *RCAN1 *transcript levels occurs specifically in CEM-C7-14 cells that are susceptible, but not in CEM-C1-15 cells which are refractory, to GC-evoked apoptosis [6]. Studies presented here further evaluate the GC-dependent regulation of individual transcripts of RCAN1 isoforms.

### Dex specifically upregulates *RCAN1-1 *transcript levels

**Figure 1:**
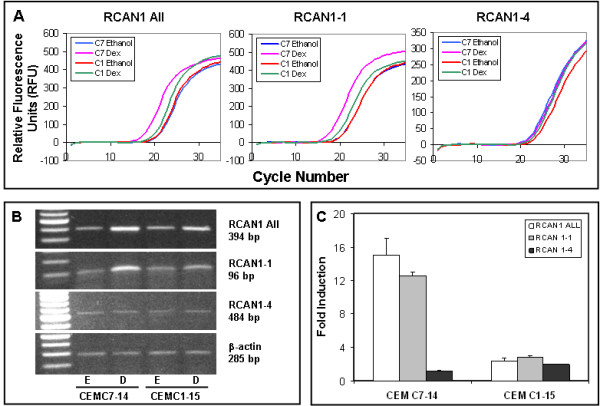
**GCs selectively upregulate *RCAN1-1 *specific transcript**: Total RNA extracted from cells treated with 100 nM Dex (D) or 0.15 ethanol (E) was subjected to reverse transcription reaction and analyzed by Real-time Q-PCR using primers specified in Table 1. **Panel A: **Raw amplification profiles of each RCAN1 specific transcript. **Panel B: **Gel electrophoresis of Real-time Q-PCR products after 18-22 cycles. **Panel C: **Relative expression of each transcript was calculated using the Pfaffl method using *β-actin *as a reference.

The expression of total *RCAN1, RCAN1-1 *and *RCAN1-4 *specific transcripts was analyzed by reverse transcription and quantitative real-time PCR of ethanol vehicle or 100 nM Dex treated CEM-C7-14 and CEM-C1-15 cells using exon 5-, 1- and 4-specific forward primers, respectively (Table 1). Representative raw amplification profiles (Figure 1A) and gels corresponding to total *RCAN1*, *RCAN1-1 *and *RCAN 1.4 *(Figure 1B) are shown. To normalize between samples, β-actin was amplified using specific primers shown in Table 1, and used as a reference. Relative expression of each transcript was calculated using a modification of the Pfaffl method [30] (Figure 1C). Total *RCAN1 *and *RCAN1-1 *transcript levels were induced 15-and 13-fold in CEM-C1-14 cells, but were not significantly altered in CEM-C1-15 cells after 24 h treatment with Dex (Figure 1C). Using a two sample t-test, the Dex response in CEM-C7-14 cells was significant compared to CEM-C1-15 cells (p < 0.015) for both total *RCAN1 *and *RCAN1-1*. Basal expression of *RCAN1-4 *was minimal in both cell types, and was not affected by Dex treatment (Figure 1).

**Table 1:** PCR Primers

**Transcript**	**Forward**	**Reverse**	**Size**
RCAN1 All	5'GGACATCACCTTTCAGTATT3'	5'TTCCTCTTCTTCCTCCTTCT3'	394 bp
RCAN1-1	5'ACCATCGCCTGTCACCTGGA3'	5'GGTGATGTCCTTGTCATACGTCCT3'	96 bp
RCAN1-4	5'CTCCCTGATTGCCTGTGTGG3'	5'TTCCTCTTCTTCCTCCTTCT3'	484 bp
β actin	5'TCATGAAGTGTGACGTTGACATCCGT3'	5'CTTAGAAGCATTTGCGGTGCACGATG3'	285 bp

RCAN1 All = total RCAN1. Forward primers for RCAN1 All, RCAN1-1 and RCAN1-4 correspond to exons 5, 1 and 4 respectively. A common reverse primer for RCAN1 All and RCAN1-4 corresponds to exon 7, while the reverse primer for RCAN1-1 corresponds to exon 5.

### Calcium signaling upregulates *RCAN1-4 *but not *RCAN1-1*

**Figure 2:**
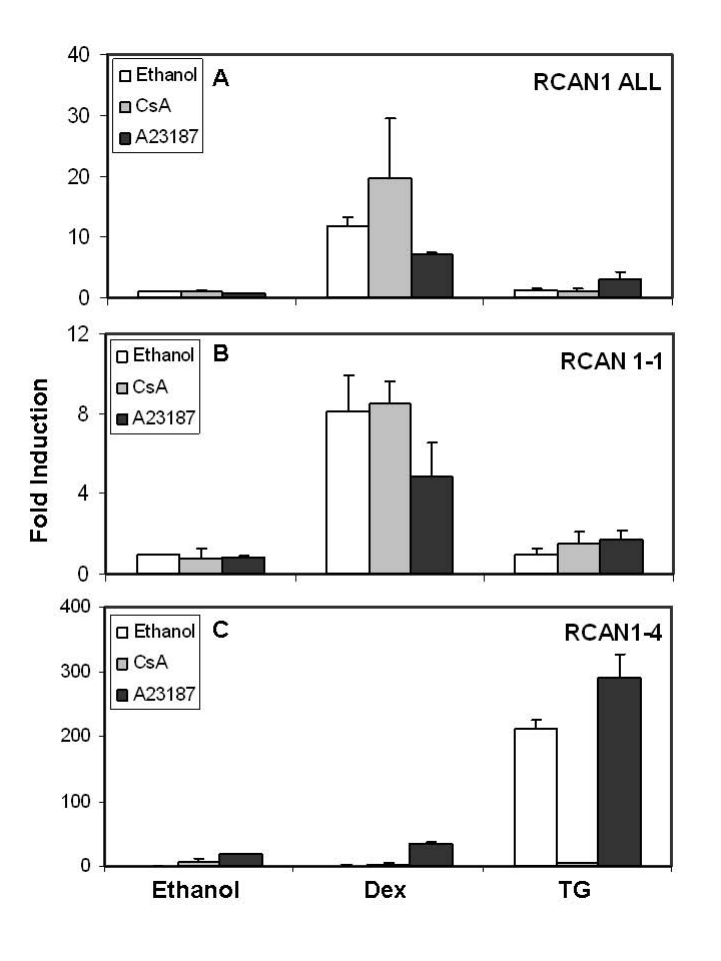
**Regulation of *RCAN1-1 *and *RCAN1-4 *expression by calcium signaling: **CEM-C7-14 cells were treated for 24 h with either ethanol vehicle, Dex or 500 nM thapsigargin (TG) in the presence or absence of the calcineurin inhibitor cyclosporin A (CsA, 600 nM) or the calcium ionophore A23187 (150 nM). RT-QPCR analysis was performed as in Figure 1 for total *RCAN1 *(RCAN1 All, **Panel A**), *RCAN1-1 *(**Panel B**) and *RCAN1-4 *(**Panel C**) using primers specified in Table 1. Data were normalized using *β-actin *as a reference, and fold induction was calculated using the Pfaffl method (28). Data presented are averages of 2-4 independent experiments.

Various reports suggest that *RCAN1 *expression is regulated by intracellular calcium ([Ca^2+^]_*i*_) levels [17,21]. Since GCs upregulate [Ca^2+^]_*i *_levels in CEM cells in correlation with apoptosis [31], we determined whether Dex-dependent upregulation of *RCAN1-1 *was modulated by calcium signaling. CEM-C7-14 cells were treated for 24 h with either Dex or thapsigargin (TG; inducer of [Ca^2+^]_*i *_levels) in the presence or absence of the calcium ionophore A23187, or the calcineurin inhibitor cyclosporin A (CsA), and expression of transcripts corresponding to total *RCAN1*, *RCAN1-1 *and *RCAN1-4 *was evaluated by RT-QPCR. *RCAN1-4 *is a minor transcript compared to *RCAN1-1*; therefore expression profiles of total RCAN1 (Figure 2A) correlated mostly with those of *RCAN1-1 *(Figure 2B) and any regulation of *RCAN1-4 *did not influence total *RCAN1 *profiles. Dex-induced upregulation of total *RCAN1 *and *RCAN1-1 *was partially inhibited by A23187 (p < 0.02 and 0.03 respectively), suggesting that Dex-dependent induction of these transcripts was inhibited by increases in [Ca^2+^]_*i *_levels (Figures 2A and 2B). CsA enhanced Dex-evoked induction of total *RCAN1*, but the effect was not statistically significant. Expression of *RCAN1-4 *was induced 200 and 18-fold, respectively by 500 nM TG and 150 nM A23187 (Figure 2C), suggesting that *RCAN1-4 *expression was regulated via the calcium signaling pathway. This was confirmed by the observation that 600 nM CsA blunted TG-dependent induction of *RCAN1-4 *(Figure 2C) (p < 0.003).

### Dex upregulates RCAN1-1 protein levels

**Figure 3:**
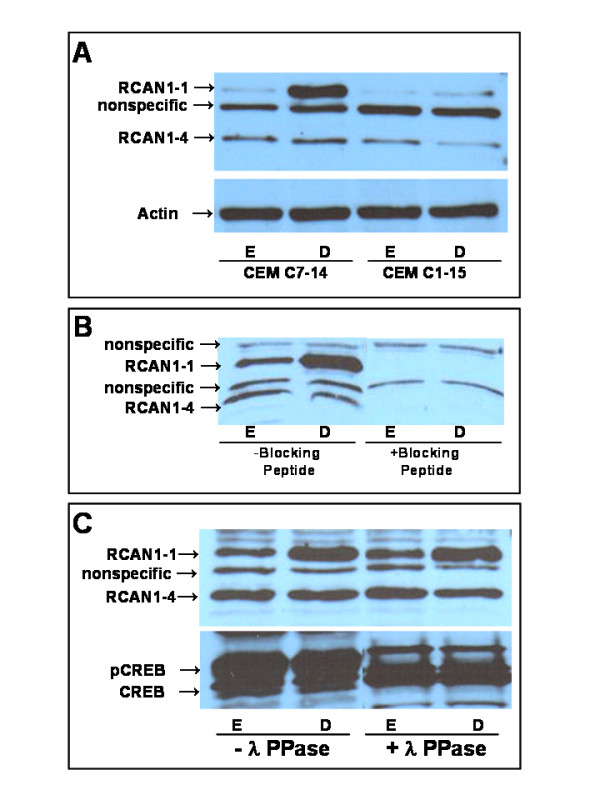
**GCs induce RCAN1-1 protein levels in CEM-C7-14 Cells: Panel A: **CEM-C7-14 and CEM-C1-15 cells were treated with ethanol vehicle (E) or 100 nM Dex (D) for 24 h. Western blotting was performed on 50 μg protein extracts using RCAN1 antibody (Abgent # AP6315c), or β-actin specific antibody (Santa Cruz # sc-8432). **Panel B: **Lysates prepared from ethanol (E) or Dex (D) treated CEM-C7-14 cells were subjected to Western blotting as in Panel A, but the antibody was preincubated for 1 h with or without blocking peptide (Abgent # BP6315c). **Panel C: **Lysates prepared from CEM-C7-14 cells treated with ethanol vehicle (E) or 100 nM Dex (D) for 24 h were treated with lambda buffer alone or lambda phosphatase for 20 min prior to Western blotting with either RCAN1 antibody as in Panel A, or CREB antibody (Santa Cruz #sc-186) to detect phosphorylation state.

Cells treated with ethanol vehicle or 100 nM Dex for 24 h were analyzed for RCAN1 protein expression by Western blotting using an antibody that recognizes the carboxyl terminal of RCAN1, and therefore detects both RCAN1-1 and RCAN1-4. Accordingly, a fast migrating minor band around 29 kDa representing RCAN1-4, and a 41 kDa band representing RCAN1-1 were detected (Figure 3A). One or two additional bands recognized by the antibody in various experiments were found to be nonspecifically interacting proteins, as demonstrated through the use of a blocking peptide (Abgent, Cat # BP6315c) specific for the antibody (Figure 3B). Preincubation of the antibody with the blocking peptide competed out the two RCAN1 bands, while not affecting the ability of the antibody to recognize the two nonspecific bands. The induction of RCAN1-1 was minimal in CEM-C1-15 cells (Figure 3A).

### Phosphorylation state of RCAN1-1

To determine whether RCAN1-1 was pohsphorylated, cell lysates were treated with lambda phosphatase prior to Western blotting (Figure 3B) to detect either RCAN1 or CREB using appropriate antibodies. CREB shifted to a faster migrating band, demonstrating a loss of phosphate residues, and confirming successful phosphatase treatment of the sample. The migration of RCAN1-1, or the relative abundance of the band was not affected by the phosphatase treatment, suggesting that phosphatase treatment did not alter its phosphorylation state. It is possible that the gel is not capable of resolving small differences in migration of phosphorylated and unphosphorylated RCAN1-1. To test this hypothesis, lysates of ethanol or Dex treated CEM-C7-14 cells were immunoprecipitated with phospho-serine, phospho-threonine or phospho-tyrosine specific antibodies coupled to Agarose beads. None of these antibodies pulled down RCAN1-1(data not shown), supporting the hypothesis that RCAN1-1 was not phosphorylated.

### Dex-evoked RCAN1-1 upregulation is kinase dependent

**Figure 4:**
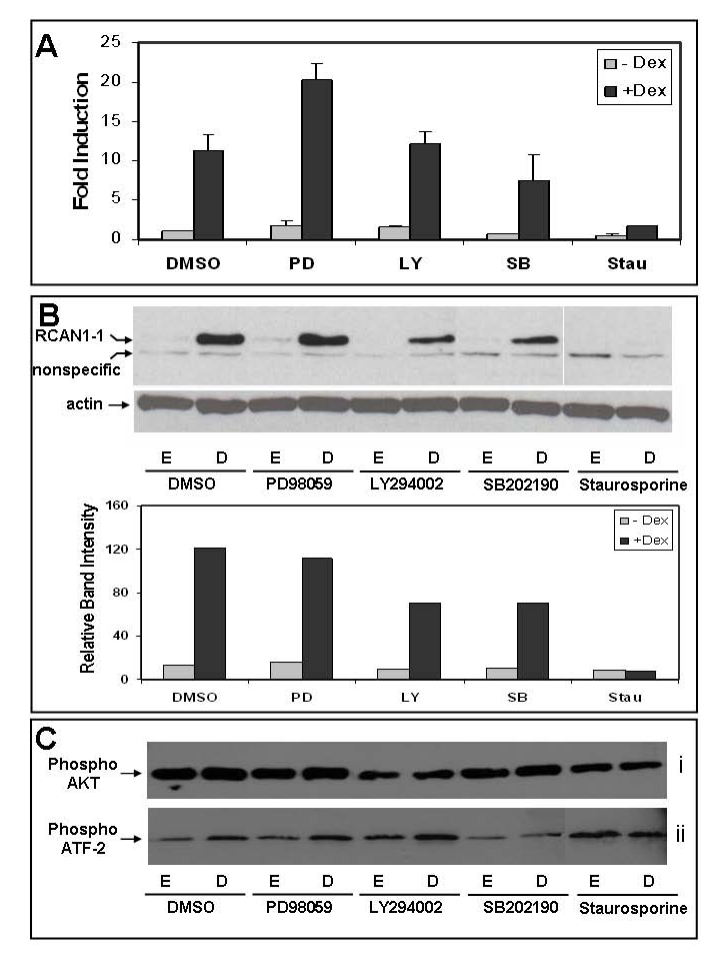
**GCs induce *RCAN1-1 *transcript and protein levels in CEM-C7-14 Cells in a kinase-dependent fashion**: **Panel A: **CEM-C7-14 cells were treated for 24 h with the indicated kinase inhibitors in the presence of ethanol (E) or 100 nM Dex (D), total RNA was extracted, and analyzed by reverse transcription-Real-time Q-PCR using primers specific for *RCAN1-1*. Relative transcript expression in various conditions was calculated by the Pfaffl method using β-actin as a reference. **Panel B: **CEM-C7-14 cells were treated with the indicated kinase inhibitors in the presence of either ethanol (E) or 100 nM Dex (D) for 24 h prior to Western blotting with either RCAN1 or β-actin specific antibodies as in Panel A. Densitometric analysis of two independent experiments was performed using ImageJ software, and average data are presented below the gel. **Panel C**: CEM-C7-14 cells were treated with the indicated kinase inhibitors in the presence of either ethanol (E) or 100 nM Dex (D) for 24 h were subjected to (i) Western blotting with phospho-Akt specific antibody (Cell Signaling Technology, cat # 9271) or (ii) p38 MAP kinase activity using a kit from Cell Signaling Technology (cat # 9820), as described in the methods section.

CEM-C7-14 cells were simultaneously treated with various kinase inhibitors in the presence of either ethanol or Dex, and processed for QPCR analysis (Figure 4A) or Western blotting (Figure 4B) to determine whether GC-mediated upregulation of *RCAN1-1 *transcript or protein was kinase dependent. The concentration for each inhibitor was close to its IC50 value: PD98059 and LY294002 were at 5 μg/ml, SB202190 was at 1 μg/ml and staurosporine was at 40 nM [32]. A strong correlation was observed between transcript and protein levels, with the exception of the MEK inhibitor, PD98059, which was shown to enhance Dex-mediated upregulation of *RCAN1-1 *transcript (p < 0.001) while not significantly affecting RCAN1-1 protein levels. The PI3 kinase inhibitor LY294002 did not significantly alter *RCAN1-1 *transcript upregulation by Dex, but reduced the intensity of RCAN1-1 protein in Western blotting to about sixty percent of Dex levels, as estimated by densitometric scanning of two independent blots (Figure 4B). The p38 MAP kinase inhibitor SB202190 partially blunted Dex-mediated upregulation of both *RCAN1-1 *transcript (p < 0.1) and protein (to 60%). Staurosporine significantly curtailed Dex-dependent expression of *RCAN1-1 *transcript (p < 0.001) and reduced protein expression to basal levels. These data suggest that Dex-dependent upregulation of *RCAN1-1 *is at least partially p38 MAP kinase dependent. Figure 4C demonstrates the specificity of the inhibitors used in inhibiting the respective kinase. Lysates prepared from CEM-C7-14 cells treated with each kinase inhibitor in the presence of either ethanol or Dex were subjected to Western blotting with an antibody specific for the PI3 kinase product phospho-Akt in Figure 4Ci. The PI3 kinase inhibitor LY294002 partially inhibited Akt phosphorylation, as depicted by the reduced intensity of bands in lysates of cells treated with 5 μg/ml of LY294002. To evaluate the specificity of p38 MAP kinase inhibition by SB202190, lysates were subjected to a p38 MAP Kinase assay using a kit (Cell Signaling Technology, cat # 9820) (Figure 4Cii). p38 MAP kinase from appropriately treated CEM-C7-14 cell lysates was immunoprecipitated with an immobilized phospho-p38 MAP kinase antibody followed by an *in vitro *kinase assay using the substrate ATF-2. Extent of phospho ATF-2 product was subsequently detected by Western blotting using a specific antibody. Figure 4Cii shows that Dex treatment induces p38 MAP kinase activity, which is blunted in the presence of 1 μg/ml SB202190.

### RCAN1-1 binds to calcineurin A

**Figure 5:**
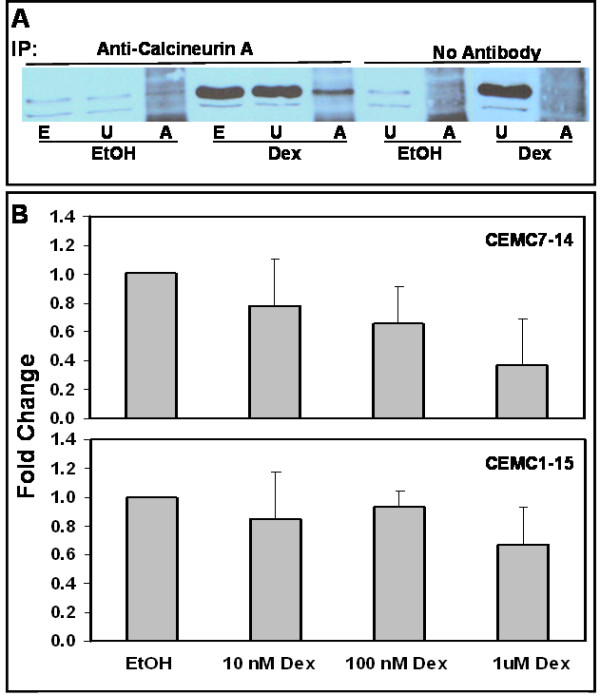
**Calcineurin binds to RCAN1-1 in correlation with loss of phosphatase activity**: **Panel A: **CEM-C7-14 cells were treated with ethanol vehicle (EtOH) or 100 nM Dex (Dex) for 24 h, cell lysates were prepared in RIPA buffer and subjected (500 μg protein) to immunoprecipitation with either anti-calcineurin A antibody or no antibody, as indicated. Unadsorbed lysates (U; 50 μg protein) or proteins bound to Protein A-Agarose (A; entire pellet) were run alongside total cell lysates (E; 50 μg protein). Western blotting was performed using RCAN1 antibody (Abgent # AP6315c). **Panel B: **CEM-C7-14 or CEM-C1-15 cells treated with 0-1 μM Dex were subjected to a calcineurin phosphatase activity assay using a kit (Biomol). Cells were lysed in the lysis buffer provided, and were cleared of any free phosphate by passing through Micro Bio-Spin P-30 Tris chromatography columns (Bio-Rad). Phosphate free lysates were incubated with a known calcineurin substrate phosphopeptide, RII, and the free phosphate released was measured using a Malachite Green based assay. Okadaic acid was used to inhibit PP1 and PP2A and Okadaic acid and EGTA was used to block PP1, PP2A and PP3C (calcineurin) activity. To calculate PP3C activity, okadaic acid inhibited activity was subtracted from total phosphatase activity. Alternatively, okadaic acid and EGTA inhibited activity was subtracted from okadaic acid inhibited activity. Phosphate released in nmoles was corrected for protein content, measured using the Bradford assay. Data are averages ± SD of two (CEM-C1-15) or three (CEM-C7-14) independent treatments, each normalized to the ethanol control.

Lysates from CEM-C7-14 cells treated for 24 h with either ethanol vehicle or 100 nM Dex were adsorbed to Protein A-Agarose conjugated to an antibody specific for calcineurin A, the beads were washed with RIPA buffer to remove non-specifically bound proteins, and subjected to Western blot analysis using an antibody specific for RCAN1-1. (Figure 5A). Since Dex treated samples had greater abundance of RCAN1-1, a greater fraction bound to and coimmunoprecipitated with calcineurin.

### Dex suppresses Calcineurin Phosphatase activity

Interactions between RCAN1 and calcineurin have been shown to inhibit calcineurin activity [19]. The abundance of RCAN1-1, and hence the proportion of calcineurin bound to RCAN1 increases in response to GC treatment of CEM-C7-14 cells (Figure 5A). In correlation, calcineurin phosphatase activity, measured by estimating the phosphate released from a phosphopeptide substrate, also decreases in a dose-dependent manner in response to Dex in CEM-C7-14 cells (Figure 5B). Calcineurin activity in cells treated with 1 μM Dex was 36% of that in ethanol treated control CEM-C7-14 cells (p < 0.03). A dose-dependent decrease in calcineurin activity in response to Dex was not apparent in CEM-C1-15 cells, however, a small decrease at 1 μM Dex (67% of control) was observed, but was not found to be statistically significant. These data suggest that RCAN1-1 binding may inactivate calcineurin. Calcineurin has been shown to protect T-cells from GC-evoked apoptosis [9], and promote T cell survival [33], hence loss of calcineurin activity may contribute to GC-evoked apoptosis.

## Discussion

Microarray analysis has correlated specific upregulation of *RCAN1 *with GC-dependent growth arrest and apoptosis of leukemic T lymphoblasts and apoptosis of pre B-leukemia cells [6,34]. RCAN1 exists as two major isomeric forms, RCAN1-1 and RCAN1-4, encoded by alternative splicing of exons 1 and 4 respectively to common exons 5-7 [16]. Differential expression of the two *RCAN1 *isoforms in response to oxidative and calcium induced stress has been reported, although both isoforms have been shown to bind to and modulate calcineurin activity [17,21,35]. Intron 3 contains 15 NFAT binding sites, which enable Ca^2+^-dependent upregulation of *RCAN1-4*; however such regulation of *RCAN1-1 *has not been reported [19]. Data presented here suggest that transcripts specific for total *RCAN1*, and isoform 1, *RCAN1-1*, are selectively upregulated by Dex in CEM-C7-14 cells, but are not significantly altered in CEM-C1-15 cells, linking *RCAN1-1 *expression with GC-evoked apoptosis. A similar selective GC-dependent upregulation of *RCAN1-1 *has been reported in correlation with apoptosis in pre B-leukemia 697 cells [22].

Interestingly, GC-evoked apoptosis of leukemic CEM-C7-14 cells is associated with induction of [Ca^2+^]_*i *_levels and is partially blocked by calcium chelators [31], suggesting that upregulation of the Ca^2+^-dependent *RCAN1-4 *may contribute to GC-evoked apoptosis. Our data suggest that Dex does not regulate RCAN1-4. Basal expression of *RCAN1-4 *is minimal in both CEM-C7-14 and CEM-C1-15 cells, and Dex concentrations known to induce [Ca^2+^]_*i *_levels selectively in CEM-C7-14 cells [31] failed to significantly alter *RCAN1-4 *expression (Figure 2C). In contrast, thapsigargin and A23187 selectively upregulated *RCAN1-4*, while having negligible effects on *RCAN1-1 *expression in CEM-C7-14 cells. Additionally, Cyclosporin A, an inhibitor of calcineruin signaling, blocked *RCAN1-4 *upregulation, but had no effect on *RCAN1-1 *expression. Differential transcriptional regulation of the two *RCAN1 *isoforms suggests that they may be involved in distinct down stream regulatory pathways, as has been reported recently [36]. RCAN1 has been reported to either inhibit or facilitate calcineurin activity under different conditions [23,24]. Calcineurin-dependent upregulation of *RCAN1-4 *has been implicated as a regulatory loop governing the extent of cellular calcineurin activity [17,20]. Perhaps Dex-evoked stimulation of RCAN1-1 and subsequent loss of calcineurin activity is sufficient to block calcineurin-dependent upregulation of *RCAN1-4 *transcription.

Phosphorylation of RCAN1 has been reported to occur primarily on two serine residues within the FLSIPP motif encoded by exon 6, with conflicting effects on calcineurin activity [16]. Phosphorylation of RCAN1-1 transfected in neuroblastoma cells enhanced its ability to inhibit calcineurin, and decreased its half-life [26]. Conversely, in cardiac myocytes and CHO-AT1 cells, RCAN1 phosphorylation at the FLISPP motif blocked its ability to bind to and inhibit calcineurin [25]. Endogenous/physiological levels of expression of RCAN1, when phosphorylated, seem to positively regulate calcineurin activity, while exogenous or over expressed RCAN1 phosphorylation may enhance negative regulation of calcineurin. Indeed, phosphorylation of one or both serine residues in a 13-amino acid synthetic peptide attenuated *in vitro *inhibition of calcineurin activity [17]. Lambda phosphatase (a potent phosphatase with specificity for phospho-Ser/phospho-Thr and phospho-Tyr) treatment of CEM-C7-14 lysates did not alter the mobility of RCAN1-1 in Western blotting experiments, suggesting no phosphatase-induced change in the phosphorylation state of RCAN1-1, or perhaps the inability of the gel to resolve phospho- vs non-phospho-RCAN1-1 into distinct bands. Further, phospho-serine, phosphor-threonine and phospho-tyrosine specific antibodies were not able to pull down RCAN1-1 in immunoprecipitation experiments, suggesting that RCAN1-1 in basal or Dex-stimulated CEM cells may not by phosphorylated, however further experiments are needed to substantiate this hypothesis.

Treatment of cells with kinase inhibitors in conjunction with Dex reduced the abundance of RCAN1-1. The broad spectrum kinase inhibitor staurosporine completely abolished Dex-dependent induction of RCAN1-1, while the p38 MAP Kinase inhibitor SB202190 partially inhibited Dex-dependent upregulation of RCAN1-1. Thus, Dex-mediated RCAN1-1 induction seems to occur via a kinase(s)-dependent pathway. This is consistent with earlier reports of a correlation between p38 MAP kinase activation, phosphorylation of GR at Ser 211, and apoptosis of CEM-C7-14 cells [37], and MAP kinase mediated induction of proapoptotic Bim in CEM-C7-14 cells [38].

The precise effect of RCAN1 on calcineurin activity is not clear. Calcineurin-dependent regulation of gene transcription plays an important role in T-cell activation and apoptosis. In one study [23] mice lacking *RCAN1 *showed altered transcription and caused aberrant expression of Fas ligand and consequent apoptosis of T helper type 1 cells during their proliferation phase, suggesting that loss of RCAN1enhances calcineurin activity. In another study, mouse embryonic fibroblasts deficient in *RCAN1 *had a disruption in the activation of calcineruin-dependent NFAT signaling, suggesting that RCAN1 was required for calcineurin function [24]. This study also reported increased apoptosis of CD4+ T cells lacking *RCAN1*, although simultaneous deletion of the *calcineurin Aβ *gene did not rescue these cells, but rather enhanced apoptosis, suggesting that RCAN1 functions as a permissive or facilitative factor for calcineurin-NFAT signaling. The data presented in these two reports regarding the effect of *RCAN1 *knockout are comparable with regard to T helper cell apoptosis, however are contradictory with regard to interpretation of the effect of *RCAN1 *on calcineurin activity. Glucocorticoids and calcineurin have been shown to inhibit each others apoptotic functions in T cell hybridomas [9], although both agents induce apoptosis. In CEM cells, our previous studies demonstrate that GC-evoked apoptosis is associated with activation of the calcium signaling pathway [31], however the calcineurin inhibitor cyclosporin A induces apoptosis and potentiates GC-evoked apoptosis (data not shown). Figure 5B demonstrates that calcineurin activity is repressed by Dex, in correlation with its accumulation in the RCAN1-1 bound form, in CEM-C7-14 cells. These observations suggest that GC-mediated upregulation of RCAN1-1 may inhibit calcineurin activity. Dex-evoked inhibition of calcineurin activity is significantly lower in CEM-C1-15 cells, which are refractory to GC-evoked apoptosis, although the low expression of RCAN1-1 protein made it difficult to determine whether RCAN1-1 interacts with calcineurin A in CEM-C1-15 cells.

## Conclusion

**Figure 6:**
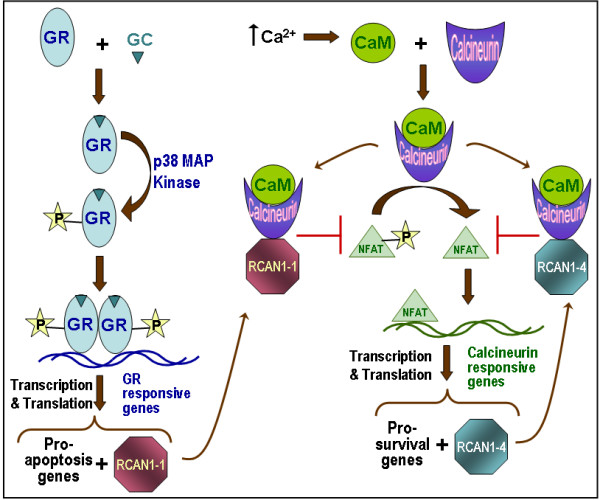
**RCAN1 mediated regulation of cell proliferation and apoptosis: **Schematic shows that GC-dependent GR activation and subsequent MAP kinase-dependent phosphorylation enables GR binding to GRE sequences on target genes. GR alters expression profiles of T-lymphoid cells resulting in a net increase in pro-apoptotic gene expression. One of the gene that GR induces is RCAN1-1. Increases in [Ca^2+^]_*i *_levels triggers the calcium signaling pathway, culminating in the activation of calcineurin phosphatase (PP3C), which dephosphorylates and activates NFAT, causing its nuclear translocation. NFAT induces transcription of pro-survival genes, and RCAN1-4, which serves as a negative feed-back regulatory loop, because it can directly bind to and inactivate calcineurin phosphatase. GR-dependent induction of RCAN1-1 also causes inhibition of calcineurin, and subsequent down-regulation of pro-survival genes, thus facilitating GR-dependent apoptosis of T lymphoid cells.

Based on data presented here, and work done by others, we conclude that, as illustrated in Figure 6, RCAN1-1 and RCAN1-4 are regulated by distinct pathways, however, have a common downstream target in PP3C, which both isoforms are capable of inhibiting. RCAN1-1 is a target of the pro-apoptotic GC-response pathway, and its expression can be blunted by inhibitors of GR activation, such as the p38 MAP kinase inhibitor SB202190 and staurosporine. By inhibiting calcineurin activity, RCAN1-1 is inhibiting expression of key survival genes, and therefore driving the cell towards apoptosis. RCAN1-4, on the other hand, seems to be a regulatory molecule which keeps PP3C activity in check.

## Methods

### Reagents

Dexamethasone (Dex), the calcium ionophore A23187, Cyclosporin A (CsA) and thapsigargin (TG) were purchased from EMD Biosciences (Madison, WI). Reagents for reverse transcription (RT) and Real-time QPCR, including M-MLV reverse transcriptase, oligo(dT)_15 _primer, RNasin^® ^Ribonuclease inhibitor, dNTP mix, and Taq DNA polymerase were purchased from Promega Life Sciences (Madison, WI). SYBR^® ^JumpStart™ *Taq *ReadyMix was from Sigma-Aldrich (St. Louis, MO). A polyclonal rabbit antibody (Cat # AP6315c) directed against the C-terminal region RCAN1 that recognizes both RCAN1-1 and RCAN 1-4 as 41 kDa and 29 kDa bands respectively, was from Abgent (San Diego, CA), phosphor-Akt(Ser473) antibody (Cat # 9271) was from Cell Signaling Technology (Beverly MA), calcineurin A antibody (Cat. #C1956) was from Sigma-Aldrich (St. Louis, MO), CREB antibody (Cat # sc-186), secondary horseradish peroxidase (HRP) conjugated anti-rabbit IgG and anti-mouse IgG were from Santa Cruz Biotechnology (Santa Cruz, CA), the ECL Western Blotting substrate was from Pierce Biotechnology (Rockford, IL). P38 MAP kinase assay kit (cat # 9820) was from Cell Signaling Technology (Beverly MA). Calcineurin Cellular Assay Kit PLUS was from BIOMOL International, Plymouth Meeting, PA. Lambda Phosphatase (Cat. # 14405) was from Upstate Biotechnology, Temecula, CA. Other reagent grade chemicals were purchased from Fisher Scientific (Pittsburgh, PA) or Sigma-Aldrich (St. Louis, MO).

### Cell Culture

Tissue culture media and components, including fetal bovine serum (FBS) were purchased from Mediatech (Washington D.C.). CEM-C7-14 and CEM-C1-15 cells were kindly provided by Dr. E. B. Thompson (UTMB, Galveston, TX), and are derivatives of the parental line CCRF-CEM, obtained from a patient with acute lymphoblastic leukemia [39]. Cells were cultured in RPMI 1640 medium supplemented with 5% FBS at 37°C in a humidified 5% CO_2 _incubator, and were maintained in log phase by passaging every 3 days. Cell treatments were for 24 h in RPMI supplemented with 5% FBS. All treatment agents were prepared as 1000× stock solutions in either ethanol or DMSO. A vehicle alone (0.1% ethanol or 0.1% DMSO) control was used in all experiments.

### Real time RT-Q-PCR analysis

Cells were treated at a density of 4 × 10^5 ^cells/ml for 24 h with the appropriate agent, and RNA was extracted from approximately 1 × 10^7 ^cells using the TRIzol reagent (Invitrogen Life Technologies, La Jolla, CA). For first-strand DNA synthesis, 5 μg of total RNA was reverse transcribed for 3 h at 42°C in the presence of 0.5 μg of oligo(dT)_15_, 1 μl (~200 U) of M-MLV reverse transcriptase, 0.5 mM dNTP mix, and 100 U of RNase inhibitor. PCR amplification of transcripts specific for total *RCAN1 *(*RCAN1 *All) and *RCAN1-4 *was accomplished using forward primers corresponding to sequences within exon 5 and exon 4, respectively, and a reverse primer corresponding to exon 7. *RCAN1-1 *was amplified using forward and reverse primers corresponding to sequences within exon 1 and 5 respectively. Primer sequences are shown in Table 1. Sequences for *RCAN1 *transcripts were extracted from the GenBank database (Accession #s NM-004414 and NM-203418). For real time QPCR, 1 μl of reverse transcription product was amplified in a Cephid SmartCycler with 125 pmoles of each primer and 12.5 μl of the SYBR^® ^JumpStart™ *Taq *ReadyMix (Sigma, cat # S-4438) in a 25 μl reaction under the following conditions: 30 sec at 94°C, 45 sec at 50°C (55°C for *RCAN1-4*), 1 min at 72°C. In duplicate reactions PCR was terminated during the exponential phase and PCR products were resolved on a 1% Agarose gel in Tris-Borate-EDTA buffer, as per conventional procedures. To normalize between samples, *β-actin *was used as a control (primers are shown in Table 1). To quantitate relative expression levels, the crossing point (CP; or cycle threshold Ct) values for each sample were used to calculate fold inductions using the Pfaffl method formula[30]: (E_target_)^ΔCPtarget^/(E_ref_)^ΔCPref ^where ΔCPtarget = (CP_ethanol_-CP_sample_) for *RCAN1 *and ΔCPref = (CP_ethanol_-CP_sample_) for *β-actin*, and the software LinRegPCR.

### Western Blotting

Cells plated at a density of 4 × 10^5 ^cells/ml were treated for 24 h with the appropriate agents, and approx. 8 × 10^6 ^cells were harvested, washed and lysed in buffer containing 50 mM Tris-HCl, pH 7.4, 150 mM NaCl, 1% NP-40, plus a protease and phosphatase inhibitor cocktail. The amount of protein in each sample was estimated using the Bradford assay, and 50 μg of each sample was boiled in SDS-PAGE sample buffer (final composition: 120 mM Tris, 4% SDS, 20% glycerol, 5% 2-mercaptoethanol, 0.05% bromophenol blue, pH 6.8) Samples were resolved on a 10% polyacrylamide-SDS gel, and electoblotted on to PVDF membranes. Membranes were blocked in 10% non-fat dry milk and incubated sequentially with a RCAN1-specific polyclonal antibody, AP6315c, and a HRP-coupled anti-rabbit secondary antibody. Membranes were developed using an Enhanced Chemiluminescence (ECL) kit from Pierce. To detect the phosphorylation state of RCAN1-1, cell lysates were treated with 400 U of lambda phophatase for 20 min prior to loading the gel.

### Immunoprecipitation

Appropriately treated CEM-C7-14 cells were washed with phosphate buffered saline (PBS, pH 7.4) and lysed with RIPA buffer (final concentration: 150 mM NaCl, 50 mM Tris pH 8.0, 1% NP-40). Calcineurin antibody was added in Protein G-Agarose (50% suspension) and incubated overnight at 4°C on a rotor. Protein G-Agarose was washed three times with RIPA buffer and cell lysate corresponding to 500 μg of proteins were added and incubated overnight at 4°C on a rotor. Protein G-Agarose was washed three times with RIPA buffer before 15.0 μL of 2× SDS PAGE reagent was added.

### p38 MAP Kinase Activity Assay

The p38 MAP kinase assay kit from Cell Signaling Technology was used to estimate the specificity of kinase inhibitors in inhibiting MAP kinase activity. Immobilized phospho-p38 MAP kinase antibody was used to pull down p38MAP kinase from 500 ug of appropriately treated CEM-C7-14 cell lysates. The Agarose beads were washed and resuspended in 25 μl kinase buffer supplemented with 200 μM ATP and 20 μg/mL of the p38 MAP kinase substrate, ATF-2 fusion protein. After incubation at 30°C for 1 h, the reaction was stopped by addition of 5× SDS-PAGE sample buffer. Western blotting was performed using ATF-2 specific antibody provided in the kit.

### Calcineurin Activity Assay

The Calcineurin cellular assay kit Plus (AK-816) from Biomol International (Plymouth Meeting, PA) was used for measurement of calcineurin activity in CEM cells in response to 0 to 1 μM Dex. Cells seeded at a density of 5 × 10^5^/ml were treated with Dex for 24 h were lysed in the manufacturer's lysis buffer containing protease inhibitors. Cell lysates were passed through size-exclusion micro Bio-Spin 30 columns (Bio-Rad, Hercules, CA) to remove free phosphate, and were incubated with RII phosphopeptide, a known substrate for calcineurin. The phosphate released was measured by a Malchite Green assay. As per guidelines provided in the kit, PP1 and PP2A activity was inhibited by okadaic acid, while EGTA and okadaic acid both were used to measure PP2C activity. Phosphate released from these two conditions was subtracted from total phosphate released in the absence of any inhibitor, to reveal PP3C activity.

## Competing interests

The authors declare that they have no competing interests.

## Authors' contributions

YH, LJN and RDM were involved in experimental design and data analysis. YH performed the experiments and helped with preparation of figures. LJN participated in critical analyses of data and provided expert technical advice. RDM prepared the manuscript and served as the principal investigator. All authors have read and approved the final manuscript.
